# Acetylcholinesterase Inhibition and *in Vitro* and *in Vivo* Antioxidant Activities of *Ganoderma lucidum* Grown on Germinated Brown Rice

**DOI:** 10.3390/molecules18066663

**Published:** 2013-06-07

**Authors:** Md. Abul Hasnat, Mehnaz Pervin, Beong Ou Lim

**Affiliations:** Department of Applied Biochemistry, College of Biomedical and Health Science, Research Institute of Inflammatory Diseases, Konkuk University, Chungju 380-701, Korea; E-Mails: noman33ju@gmail.com (M.A.H.); mehnaz.pervin@gmail.com (M.P.)

**Keywords:** *Ganoderma lucidum*, acetylcholinesterase inhibition, antioxidant potential, cellular antioxidant activity, anti-hemolysis activity

## Abstract

In this study, the acetylcholinesterase inhibition and *in vitro* and *in vivo* antioxidant activities of *Ganoderma lucidum* grown on germinated brown rice (GLBR) were evaluated. In antioxidant assays *in vitro*, GLBR was found to have strong metal chelating activity, DPPH, ABTS, hydroxyl and superoxide radical scavenging activity. Cell-based antioxidant methods were used, including lipid peroxidation on brain homogenate and AAPH-induced erythrocyte haemolysis. In antioxidant assays *in vivo*, mice were administered with GLBR and this significantly enhanced the activities of antioxidant enzymes in the mice sera, livers and brains. The amount of total phenolic and flavonoid compounds were 43.14 mg GAE/g and 13.36 mg CE/g dry mass, respectively. GLBR also exhibited acetylcholinesterase inhibitory activity. In addition, HPLC analyses of GLBR extract revealed the presence of different phenolic compounds. These findings demonstrate the remarkable potential of GLBR extract as valuable source of antioxidants which exhibit interesting acetylcholinesterase inhibitory activity.

## 1. Introduction

Oxidative stress can be defined as a state of imbalance towards the factors that generate reactive oxygen radicals (e.g., superoxide or hydroxyl radicals) and away from the factors that protect cellular macromolecules from these reactants, including antioxidants like superoxide dismutase, catalase, and glutathione peroxidases. The factors that generate reactive oxygen species (ROS) exist as products of normal cellular physiology as well as from various exogenous sources [1]. Prolonged state of oxidative stress is involved in the pathogenesis of several diseases, such as cardiovascular disease and cancer [2]. Antioxidants can prevent oxidative stress and its deleterious effects. They exert their effects by scavenging ROS or preventing their production. To act against deleterious oxidative-induced reactions, food and pharmaceutical products are normally supplemented with synthetic antioxidants such as butylated hydroxyanisole (BHA), butylated hydroxytoluene (BHT), *tert*-butylhydroquinone (TBHQ) and propyl gallate (PG). However, concerns about the long-term safety and negative consumer perception of synthetic antioxidants have led to an increasing demand for natural antioxidants [3].

Oxidative stress is also implicated in the development of many neurodegenerative diseases including Parkinson’s disease (PD), Huntington’s disease, amyotrophic lateral sclerosis and Alzheimer’s disease (AD) [4]. One of the important strategies for treating of AD is to maintain the levels of acetylcholine through the inhibition of acetylcholinesterase (AChE) [5]. A wide range of plant compounds with this inhibitory activity has been found, mainly alkaloids, xanthones and flavonols, such as quercetin, among others. Interestingly, intake of polyphenols through diets was stated to reduce incidence of certain age related neurological disorders including macular degeneration and dementia [6].

Phenolic compounds are by far the most abundant antioxidants in most human diets. Phenolic compounds possess strong antioxidant properties that enable them to scavenge free radicals, donate hydrogen, chelate metal ions, break radical chain reactions, and quench singlet oxygen *in vitro* and *in vivo* [7]. Mushrooms accumulate a variety of bioactive metabolites (e.g., phenolic compounds, polyketides, terpenes, steroids, and polysaccharides) with immunomodulatory, cardiovascular, liver protective, anti-fibrotic, anti-inflammatory, anti-diabetic, anti-viral, antimicrobial and antitumour properties [8].

*Ganoderma lucidum*, a basidiomycete white rot fungus belonging to the family Ganodermataceae, has been widely used for medicinal purposes to promote health and longevity in China, Japan and Korea for thousands of years [9]. The mushroom is considered to be a popular folk medicine for prevention or treatment of various diseases including hepatitis, hypertension, hypercholesterolemia, gastric cancer, arthritis and bronchitis. *Ganoderma lucidum* is not easily available for food in large amounts because of its high production cost and difficult growing techniques. From this point of view, we grew *G. lucidum* on germinated brown rice which is known to have rich nutrients [10]. However, germination can cause significant changes in the biochemical characteristics and during this process storage proteins can be degraded by proteases. It can increase its nutritional value, because it can improve protein digestibility, reduce antinutritional factors, and hydrolise oligosaccharides (raffinose and stachyose) [11]. It can also lead to modification of bioactive constituents as previously reported [12].

The main objective of the present study was to evaluate the antioxidant and acetylcholinesterase inhibition activity of the extract of *Ganoderma lucidum* grown on germinated brown rice (GLBR) through *in vitro* models. In addition, the potential antioxidant activity *in vivo* of GLBR extract was evaluated by measuring the changes in activities of antioxidant enzymes in mice. The presence of bioactive components was also measured by high performance liquid chromatography (HPLC).

## 2. Results and Discussion

### 2.1. Total Phenolic, Flavonoid, Ascorbic Acid, β-Carotene, Lycopene Content & Yield of GLBR Extract

Methods of extraction are very important for obtaining extracts with acceptable yields and strong antioxidant activity. In this experiment, the yield of water extract of GLBR was 24%. Phenolic compounds are secondary plant metabolites with beneficial biological effects that can scavenge free radicals. The total phenolic content of the GLBR extract was determined from a calibration curve and expressed as Gallic Acid Equivalents (GAE). The obtained values were 43.14 ± 1.29 mg GAE per gram of dry mass.

The content of flavonoid compounds of the extracts was determined from a calibration curve and expressed in Catechin Equivalents (CE). The flavonoid contents of the water extract was 13.36 ± 0.44 mg CE per gram of dry mass.

Important amount of ascorbic acid was found in GLBR extract. The obtained value was 3.8 ± 0.17 mg ascorbic acid per gram of dry mass. β-Carotene and lycopene were only found in vestigial amounts. The obtained values for β-Carotene and lycopene were 1.31 ± 0.06 µg and 0.79 ± 0.03 µg/g, respectively.

**Table 1:** Phenolic composition of *Ganoderma lucidum* grown on germinated brown rice (GLBR) extract by HPLC analysis.

Phenolic compound in GLBR	Composition (mg/mL)
Quercetin	13.53
Ursolic acid	12.87
Keampferol	8.62
Terpeniol	7.35
Coumarin	5.65
Catechin	4.09
Myricetin	3.85
Ferulic acid	2.13
Thymol	0.67
Rutin	0.65
Lupane	0.29
Cyanidin	0.04
Elagic acid	0.001
Caffeic acid	nd
Epicatechin	nd

nd: not detected.

### 2.2. HPLC Analysis of GLBR Extract

Identification of the investigated compounds was carried out by comparison of their retention time and UV spectra with those obtained injecting standards under the same conditions. In the present study, fifteen components were analysed and thirteen components were determined among them. By using the calibration curve of each investigated compound, the contents of the thirteen compounds of GLBR were determined (Table 1). The thirteen components were identified as quercetin, ursolic acid, keampferol, terpeniol, coumarin, catechin, myricetin, ferulic acid, thymol, rutin, lupine, cyaniding and elagic acid. The analysis showed that quercetin (13.53 mg/mL) and ursolic acid (12.87 mg/mL) are the major component in the water extract of GLBR. Quercetin is a strong antioxidant and radical scavenger which is abundant in fruits and vegetables [13]. Ursolic acid (UA) is also one of the major components of certain medicinal plants which possess a wide range of biological effects, such as anti-oxidative, anti-tumour, anti-inflammatory activities and neuroprotective effect [14].

### 2.3. Antioxidant Activity in Vitro

#### 2.3.1. DPPH Radical Scavenging Activity of GLBR Extract

1, 1-Diphenyl-2-picrylhydrazyl (DPPH) is a stable free radical that accepts an electron or hydrogen radical to become a stable diamagnetic molecule. The dark colour of the DPPH radical solution becomes lighter when it is mixed with an antioxidant. The degree of discoloration indicated the scavenging potential of the antioxidant compounds or extracts in the term of hydrogen or electron donating ability. The extract of GLBR showed DPPH radical scavenging activities in a dose-dependent manner. The scavenging activity ranged from 19.32% to 88.32% for GLBR extract concentrations from 0.125 to 2.0 mg/mL. The concentration of sample at which the inhibition percentage reaches 50% is defined as the IC_50_ value. IC_50_ value was 0.64 mg/mL (Table 2).

**Table 2:** DPPH, ABTS, nitrite, reducing power and metal chelating activities of GLBR extract.

**Extract**	**DPPH radical scavenging activity**	**ABTS radical scavenging activity**	**Hydroxyl radical scavenging activity**	**Superoxide radical scavenging activity**	**Metal chelating activity**	**Lipid peroxidation**	**Cellular antioxidant activity**
	IC^a^_50_ value mg/mL	IC^a^_50_ value mg/mL	IC^a^_50_ value mg/mL	IC^a^_50_ value mg/mL	IC^a^_50_ value mg/mL	IC^a^_50_ value mg/mL	IC^a^_50_ value mg/mL
GLBR	0.64 ± 0.02 ^a^	0.44 ± 0.02 ^a^	0.164 ± 0.017 ^a^	0.62 ± 0.05 ^a^	0.84 ± 0.01 ^a^	1.48 ± 0.13 ^a^	1.46 ± 0.17 ^a^
Positive control	0.19 ± 0.002 ^b^ (Ascorbic Acid)	0.02 ± 0.0002 ^b^ (Ascorbic Acid)	0.005 ± 0.0003 ^b^ (Ascorbic Acid)	0.053 ± 0.002 ^b^ (Ascorbic Acid)	0.009 ± 0.0002 ^b^ (EDTA)	0.59 ± 0.06 ^b^ (Ascorbic Acid)	0.11 ± 0.02 ^b^ (Ascorbic Acid)

Data are mean ± SD (*n =* 3). Values denoted by different letters (^a−b^) in a column are significantly different (*p <* 0.05); ^a^ IC_50_ value (mg/mL): the concentration in which 50% is inhibited.

Furthermore, DPPH radical scavenging activity was highly correlated with phenolic and flavonoid contents (r^2^ = 0.9384 and 0.9393, respectively). These analyses indicate that there is a linear relationship between antioxidant activity and total phenolic and flavonoid contents. The same correlation between antioxidant activity in the DPPH assay and total phenolic content in the mycelium of *Ganoderma lucidum* was reported by Heleno *et al.* [4]. The scavenging activity of ascorbic acid was significantly stronger than that of extract (*p <* 0.05; Table 2).

#### 2.3.2. ABTS Radical Scavenging Activity of GLBR Extract

The 2, 2′-azino-bis(3-ethylbenzothiazoline-6-sulphonic acid) diammonium salt (ABTS) radical cation decolourization test is another method widely used to assess antioxidant activity. The reaction between ABTS and potassium persulphate generates ABTS radicals. When mixed with an antioxidant, an electron is donated to the ABTS radical which is converted to a non-radical form. Colour reduction indicates reduction of the ABTS radical. GLBR extract showed significant ABTS radical scavenging activity. The scavenging activity ranged from 19.98% to 98.90% for concentrations from 0.125 to 2.00 mg/mL. The IC_50_ value was 0.44 mg/mL (Table 2). Neither the GLBR extract nor the ascorbic acid showed any significant difference in ABTS radical scavenging activity (at 2 mg/mL; *p <* 0.05). The antioxidant activity in the ABTS assay was also strongly correlated with phenolic and flavonoid contents (r^2^ = 0.8387 and 0.8401, respectively). The high phenolic content of GLBR extract might be responsible for the increased ABTS radical scavenging activity. Our results are well consistent with the previous report [15].

#### 2.3.3. Hydroxyl Radical Scavenging Activity

The hydroxyl radical is the most reactive free radical and is formed from a superoxide anion and hydrogen peroxide in presence of metal ions such as copper or iron. The hydroxyl radical scavenging effect of GLBR extract generated in the Fenton reaction was studied using the Electron Spin Resonance (ESR) technique. The extract showed dose dependent hydroxyl radical scavenging activity. The scavenging activity ranged from 46% to 75.29% for concentrations from 0.125 to 2.0 mg/mL. Ascorbic acid showed 86.97% scavenging activity at 2 mg/mL with IC_50_ value of 0.005 mg/mL which was significantly lower than that of the GLBR extract (0.164 mg/mL, *p <* 0.05; Table 2).

#### 2.3.4. Superoxide Radical Scavenging Activity

Superoxide anions are a precursor to active free radicals that have the potential for reacting with biological macromolecules and thereby inducing tissue damage. Superoxides have also been observed to directly initiate lipid peroxidation [16]. The superoxide anion plays an important role in the formation of other ROS such as hydrogen peroxide, hydroxyl radical, and singlet oxygen, which induce oxidative damage in lipids, proteins, and DNA. The spin adduct of the superoxide free radical with 5, 5′-dimethylpyrroline-1-oxide (DMPO) was detected by ESR. In this experiment, extract of GLBR showed superoxide radical scavenging activity which ranged from 19.61% to 63.61% at concentrations of 0.125 to 2 mg/mL. Ascorbic acid showed 76.13% scavenging activity at 2 mg/mL with IC_50_ value of 0.053 mg/mL which was significantly lower than that of the GLBR extract (0.62 mg/mL, *p <* 0.05; Table 2).

#### 2.3.5. Effect of GLBR Extract on Metal Chelating Activity

Transition metals have been proposed as the catalysts for the initial formation of radicals. Chelating agents may stabilize transition metals in living systems and inhibit the generation of radicals, consequently reducing free radical-induced damage [17]. To better estimate the antioxidant potential of the extract, its chelating activity was evaluated against Fe^2+^. The extract of GLBR showed chelating activity in a dose dependent manner. The scavenging activity ranged from 29.84% to 62.64% at concentrations from 0.125 to 2 mg/mL. However, the metal chelating activity of positive control ethylenediaminetetraacetic acid (EDTA) was found to be 90.87% (at 0.1 mg/mL). The observed IC_50_ value of the GLBR extract (0.84 mg/mL) was significantly higher than that of the positive control EDTA (0.009 mg/mL; *p <* 0.05; Table 2).

#### 2.3.6. Effect of GLBR Extract on Lipid Peroxidation

In biological systems, lipid peroxidation generates a number of degradation products and is found to be an important cause of cell membrane destruction and cell damage [18]. Malondialdehyde (MDA) is one of the major products of lipid peroxidation, which can be detected using a thiobarbituric acid colour reaction. GLBR extract showed dose dependent inhibition of Fe^2+^ induced lipid peroxidation in mouse brain homogenates. The inhibition activity ranged from 19.89% to 55.98% for GLBR extract concentrations from 0.125 to 2.0 mg/mL. IC_50_ value was 1.48 mg/mL (Table 2). This result is well consistent with the previous report [19].

Metal (Fe^2+^) chelating and hydroxyl radical scavenging activity were determined to explain the mechanism through which the GLBR extract inhibited Fe^2+^-induced lipid peroxidation. Metal chelating and hydroxyl radical scavenging abilities are strongly correlated with inhibition of lipid peroxidation (r^2^ = 0.9723 and 0.9147, respectively). The interactions between iron and hydrogen peroxide (Fenton’s reaction) generate the highly reactive hydroxyl radical, which initiates a process of membrane lipid peroxidation that could lead to alterations in cell structure and function [20].

#### 2.3.7. Effect of GLBR Extract on Cellular Antioxidant Activity (CAA)

The CAA assay is based on the ability of reactive oxygen species (ROS) to react with a fluorescent probe in the cell and on the prevention of this reaction in the presence of antioxidant compounds inside the cell. The DCFH-DA is a commonly used dye in biological systems. We determined the ability of the GLBR extract to scavenge free radicals by implementing the CAA assay previously described by Wolfe and Liu [21]. A significant dose-dependent inhibition was observed ranged from 5.64% to 53.22% for GLBR extract concentrations from 0.125 to 2.0 mg/mL. IC_50_ value was 1.46 mg/mL (Table 2). Cellular antioxidant activity was also strongly correlated with phenolic and flavonoid contents (r^2^ = 0.8631 and 0.8641, respectively).

#### 2.3.8. Effect of GLBR Extract on Inhibition of Erythrocyte Haemolysis

**Figure 1:**
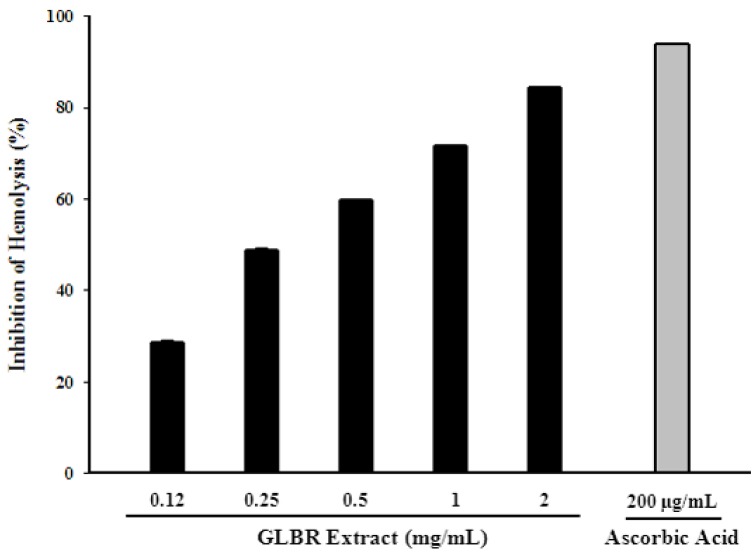
Hemolysis inhibitory activity of *Ganoderma lucidum* grown on germinated brown rice (GLBR) extract.

The oxidative hemolysis in erythrocytes induced by 2, 2′-azo-bis(2-amidinopropane) dihydrochloride (AAPH) has been studied as a model for peroxidative damage in biomembranes. Polyunsaturated fatty acids rich RBC membranes are very susceptible to lipid peroxidation induced by ROS and free radicals, which ultimately leads to haemolysis. The protective effects of GLBR extract and ascorbic acid on the hemolysis induced by AAPH are shown in Figure 1. IC_50_ values of the GLBR extract and ascorbic acid were 275.57 ± 10.52 and 17.96 ± 0.27 μg/mL, respectively. This result is in accordance with previous report [2]. However, our results showed that inhibition of lipid peroxidation and anti-haemolysis activity of GLBR extract are strongly correlated (r^2^ = 0.9365).

### 2.4. Antioxidant Activities *in Vivo*

Excessive generation of ROS or free radicals can lead to cell and tissue damage paralleled by alterations in the function of genetic apparatus, resulting in aging and untimely cell death. Superoxide dismutase (SOD), catalase (CAT) and glutathione peroxidase (GPx) belong to the main defence antioxidants that prevent the formation of new free radical species. SOD converts superoxide into hydrogen peroxide whereas GPx and CAT changes hydrogen peroxide into harmless molecules. Thus they prevent superoxide and hydrogen peroxide from interacting and producing hydroxyl and singlet oxygen through Fe-dependent Fenton Reaction. In the present investigation GLBR increased the levels of antioxidant enzymes in serum, liver and brain tissues of mice by reducing the oxidative stress due to its potential antioxidant activity and these results agree with the previous studies on antioxidants [22]. GLBR treatment enhanced the antioxidant enzymes (SOD, CAT and GPx) activity in a dose-dependent manner. Effects of GLBR extract and VC on the activities of SOD, CAT and GPx in serums, livers and brains of mice are shown in Table 3. For most of the samples tested, GLBR extract at the dose of 200 and 400 mg/kg body weight treatment groups significantly increased the activities of antioxidant enzymes as compared to control group (*p <* 0.05). In most of the cases, compared with control group, activities of antioxidant enzymes had no significant variance for the GLBR extract at the dose of 100 mg/kg body weight treatment group (*p <* 0.05). It is possible that the effect of GLBR on SOD, CAT and GPx was associated with its induction on the expression of genes of the antioxidant enzymes. The enhanced activities of antioxidant enzymes may provide an effective defence from the damaging effects of free radicals.

**Table 3:** Effects of *Ganoderma lucidum* grown on germinated brown rice (GLBR) on the activities of SOD (U/mg protein), GPx (U/mg protein) and CAT (U/mg protein) in serums, livers and brains of mice.

Group	Serum			Liver			Brain		
	SOD (U/mL)	GPx (U/mL)	CAT (U/mL)	SOD (U/mL)	GPx (U/mL)	CAT (U/mL)	SOD (U/mL)	GPx (U/mL)	CAT (U/mL)
Control	43.27 ± 5.03 ^a^	48.98 ± 4.67 ^a^	13.2 ± 0.70 ^a^	35.3 ± 2.04 ^a^	52.5 ± 4.30 ^a^	14.4 ± 0.65 ^a^	98.41 ± 5.55 ^a^	262.67 ± 8.12 ^a^	5.67 ± 0.99 ^a^
Positive control	61.53 ± 2.93 ^b^	51.69 ± 2.52 ^a^	14.7 ± 0.77 ^a^	41.7 ± 2.49 ^a^	61.7 ± 3.90 ^b^	16.3 ± 0.79 ^b^	110.45 ± 4.65 ^b^	276.07 ± 11.05 ^a^	7.27 ± 1.03 ^a^
Extract (100 mg/Kg)	44.25 ± 2.23 ^a^	49.79 ± 3.03 ^a^	14.1 ± 0.95 ^a^	31.7 ± 3.11 ^a^	65.1 ± 4.30 ^c^	16.9 ± 0.80 ^c^	123.41 ± 4.39 ^c^	275.94 ± 6.91 ^a^	7.82 ± 1.06 ^b^
Extract (200 mg/Kg)	57.56 ± 5.15 ^c^	53.08 ± 2.44 ^a^	15.7 ± 0.61 ^b^	52.8 ± 4.37 ^b^	70.5 ± 2.57 ^d^	18.1 ± 0.94 ^d^	127.64 ± 7.16 ^d^	294.35 ± 8.05 ^b^	9.93 ± 1.04 ^c^
Extract (400 mg/Kg)	71.72 ± 8.18 ^d^	60.05 ± 2.36 ^b^	17.5 ± 0.74 ^c^	67.3 ± 4.20 ^c^	79.4 ± 3.84 ^e^	19.9 ± 0.46 ^e^	138.38 ± 7.11 ^e^	305.55 ± 6.66 ^c^	11.29 ± 0.98 ^d^

Data are mean ± SD of 5 mice. Values denoted by different letters (^a−e^) in a column are significantly different (*p* < 0.05) compared to the control group.

### 2.5. Acetylcholinesterase (AChE) Inhibitory Activity

**Figure 2:**
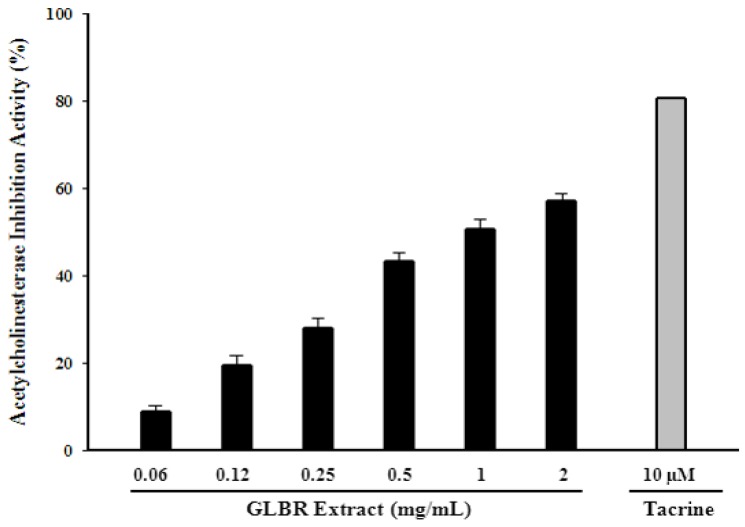
Acetylcholinesterase (AChE) inhibitory activity of *Ganoderma lucidum* grown on germinated brown rice (GLBR) extract.

Acetylcholinesterase (AChE) inhibitors have been widely used in the treatment of neurodegenerative disease. GLBR extract showed significant acetylcholinesterase inhibition activity. The activity ranged from 19.46% to 57.01% for concentrations from 0.125 to 2.00 mg/mL (Figure 2). 

The IC_50_ value was 1.01 mg/mL. Tacrine (positive control) showed 80.62% scavenging activity at 10 µM which was significantly lower than that of the GLBR extract (*p* < 0.05). The acetylcholinesterase inhibition activity was also highly correlated with total phenolic and flavonoid contents (r^2^ = 0.7736 and 0.7755, respectively). Quercetin and ursolic acid are the main components of GLBR water extract. These compounds are known to have a lot of biochemical activities. Among these, protection against neurodegenerative disease can be mentioned [13,14].

## 3. Experimental

### 3.1. Chemicals and Materials

Folin–Ciocalteu reagent (FC reagent), gallic Acid, sodium nitrite, trichloroacetic acid (TCA), ascorbic acid (AA), 1,1-diphenyl-2-picrylhydrazyl (DPPH), potassium persulphate, 2,2′-azino-bis(3-ethylbenzothiazoline-6-sulphonic acid)diammonium salt (ABTS), anhydrous sodium phosphate (dibasic), anhydrous sodium phosphate (monobasic), ferrous chloride, ammonium thiocyanate, ethylenediaminetetraacetic acid (EDTA), 5,5′-dimethylpyrroline-1-oxide (DMPO), pyrogallol, acetylthiocholine iodide (ATCI), acetylcholinesterase (AChE), 5,5′-dithio-bis(2-nitrobenzoic acid) (DTNB), tacrine (9-amino-1,2,3,4-tetrahydroacridine hydrochloride hydrate), thiobarbituric acid (TBA), malondialdehyde (MDA), dimethyl sulfoxide (DMSO), 2′,7′-dichlorofluorescin diacetate (DCFDA), 2,2′-azo-bis(2-amidinopropane) dihydrochloride (AAPH) and ferrous sulphate (FeSO_4_) were purchased from Sigma Chemical Co. (St. Louis, MO, USA). The catalase, SOD and GPx assay kit were purchased from Cayman Chemical Company (Ann Arbor, MI, USA). Dulbecco’s Modified Eagle’s Medium (DMEM) was purchased from WElGENE Inc. (Daegu, Republic of Korea). Potato Dextrose agar (PDA) media was purchased from Becton, Dickinson and Company (Sparks, MD, USA). All other chemicals were purchased from Sigma, unless otherwise specified. 

### 3.2. Preparation of Ganoderma lucidum Grown on Germinated Brown Rice (GLBR) Extract

*Ganoderma lucidum* grown on germinated brown rice were obtained as previously described [23]. Briefly, mycelia of *Ganoderma lucidum* were inoculated in PDA media for 3 weeks at 25 °C. Then mycelia from PDA media were inoculated on germinated brown rice at 20–25 °C for 5 weeks. Then cultured material was ground to a fine powder with a grinder. The powder was extracted at 85 °C with water for 3 h. Extraction was performed three times and the extracts filtered through Whatman No.1 filter paper (GE Healthcare UK Ltd., Buckinghamshire, UK). The extract was dried by a rotary evaporator under vacuum at 40 °C and freezing dried at –70 °C then stored in a freezer at –20 °C until use.

### 3.3. Measurement of Total Phenolic, Flavonoid, Ascorbic Acid, β-carotene, Lycopene Content

The total phenolic content was determined by using Folin–Ciocalteu colorimetric method with little modification [24]. Briefly, each extract (10 mg) was dissolved in DW (1 mL) and different concentrations of gallic acid (0.0078–1 mg/mL) were prepared in water. Sample (40 μL), 1 M Folin-Ciocalteu reagent (20 μL) and 20% (w/v) sodium carbonate (Na2CO3, 60 μL) were mixed. Mixtures were kept in the dark at room temperature for 30 min. Absorbance was measured at 700 nm using a UV-visible spectrophotometer. Total phenolic content was determined from the standard calibration curve. Results were expressed as mg of gallic acid equivalents (GAE) per gram of the extract.

Total flavonoid contents in the extracts were determined by a colorimetric method with some modifications [24]. The concentrations of the sample and standard (catechins) solutions were 10 mg/mL and 0.015–1 mg/mL, respectively. Briefly, each sample or standard reagent (25 μL) was mixed with DW (125 μL) and 5% sodium nitrate solution (8 μL) was added. After 5 min incubation, 10% (w/v) aluminium chloride solution (15 μL) was mixed in and the solution and allowed to stand for 6 min. Then NaOH (0.1 M, 50 μL) and Dw (27 μL) were added to the mixture. After 15 min incubation at RT, the absorbance was measured at 517 nm. Catechin was used to plot the standard curve and the results were expressed as mg of catechins equivalent (CE) per gram of the extract.

Ascorbic acid was determined according to the method of Barros *et al.* [25]. Each dried extract (100 mg) was extracted with 10 mg/ mL metaphosphoric acid (10 mL) for 45 min at room temperature and filtered through disposable filtrate (0.45 µm, Millipore, Indianapolis, IND, USA). The filtrate (1 mL) was mixed with 2, 6-dichloroindophenol (0.05 mM, 9 mL) and the absorbance was measured at 515 nm against blank. Content of ascorbic acid was calculated on the basis of the calibration curve of authentic L-ascorbic acid and the results were expressed as mg of ascorbic acid per gram of dry weight.

β-Carotene and lycopene were determined by the method of Barros *et al.* [25]. The dried extract (100 mg) was vigorously shaken with acetone-hexane mixture (4:6, 10 mL) for 5 min and filtered through a disposable filter (0.45 µm, Millipore). The absorbance of the filtrate was measured at 453, 505, and 663 nm. Contents of β-carotene and lycopene were calculated according to the following equations: β-carotene (mg/100 mL) = 0.216 (A663) − 0.304 (A505) + 0.452 (A453); lycopene (mg/100 mL) = −0.0458 (A663) + 0.372 (A505) − 0.0806 (A453). The assays were carried out in triplicates, the results were mean ± SD and expressed as µg of carotenoid/g of extract.

### 3.4. High Performance Liquid Chromatography (HPLC) Profiling of the Extract

HPLC analysis was performed by a Shimadzu UFLC system (Shimadzu Corporation, Tokyo, Japan) consisting of a vacuum degasser, an autosampler, and a binary pump equipped with a reversed-phase C18 analytical column (4.6 × 250 mm, 5 μm particle size, COSMOSIL 5C18-AR-II packed column). All solvents were filtered with a 0.45 μm filter disk. A gradient elution was carried out using the following solvent system: mobile phase A, water/acetic acid (99:1, v/v); mobile phase B, mobile phase A/acetonitrile (60:40, v/v). The gradient program was as follows: from 2 to 6% B in 16 min, from 6 to 10% B in 4 min, from 10 to 17% B in 4 min, from 17 to 36% B in 14 min, from 36 to 38.5% B in 2 min, from 38.5 to 60% B in 13 min, from 60 to 100% B in 5 min, and from 100 to 2% B in 2 min. A 10 min re-equilibration time was used after each analysis. The flow rate used was set at 0.50 mL/min throughout the gradient. The column temperature was maintained at 25 °C, and the injection volume was 10 μL. The spectra were acquired in the range of 190–400 nm and chromatograms plotted at 280 nm. Phenolic compounds were identified by matching the retention time and their spectral characteristic against those of prepared standards.

### 3.5. Assay of Antioxidant Activity *in Vitro*

#### 3.5.1. DPPH Radical-Scavenging Activity

DPPH radical-scavenging activities of ethanol and water extracts were determined by the method of Jeong *et al.* [26]. Different concentrations (0.12–2.00 mg/mL) of sample and BHT (as positive control) were prepared in water and methanol, respectively. Each sample or standard solution (80 μL) was mixed with DPPH solution (0.3 mM in methanol, 80 μL). Then, the mixture was shaken vigorously and left to stand for 30 min in the dark. Absorbance of the solution was read at 517 nm against the blank. Controls were prepared in a similar way as for the test group except for the replacement of the test sample with the corresponding extraction solvent. DPPH radical-scavenging activity was calculated by using the following equation:

Scavenging activity (%) = (1 − Absorbance of sample/Absorbance of control) × 100

#### 3.5.2. ABTS Radical-Scavenging Activity

ABTS was dissolved in water to make 7 mmol/L concentration and ABTS^+^ was produced by the reaction of ABTS stock solution with a potassium persulphate solution (at 2.45 mmol/L final concentration). The mixture was kept under dark at RT for 12–16 h before use [26]. This radical form remains stable for more than 2 days at room temperature. Freshly prepared ABTS+ solution was diluted with 0.01 M phosphate buffer saline (PBS, pH 7.4) and absorbance was adjusted to 0.70 ± 0.02 at 734 nm. Then various concentrations of the sample and ascorbic acid standard (0.12–2.00 mg/mL, 0.3 mL) were mixed with ABTS^+^ solution (0.7 mL). Finally, the absorbance was measured at 734 nm against the blank after 5 min of reaction at RT. The controls contained the extraction solvent instead of the test sample. The scavenging activity of ABTS free radical was calculated as:

Scavenging activity (%) = (1 − Absorbance of sample/Absorbance of control) × 100

#### 3.5.3. Hydroxyl Radical Scavenging Activity

Hydroxyl radicals scavenging activity was measured by JES-FA100 ESR spectrometer (JEOL Ltd., Tokyo, Japan). Hydroxyl radicals were produced by Fenton reaction and reacted rapidly with the nitrone spin trap DMPO [27]. The resultant DMPO-OH compound was detectable with an ESR spectrometer. Briefly, 0.3 M DMPO and 10 mM H_2_O_2_ were dissolved in 0.1 M phosphate buffer solution (pH 7.4). Then different concentrations (0.12–2.00 mg/mL) of sample or standard (ascorbic acid) (20 μL) were mixed with DMPO (20 μL), FeSO_4_ (10 mM, 20 μL) and H_2_O_2_ (20 μL). The ESR spectrum was recorded after 2.5 min incubation at RT. ESR spectrometer was set at the following conditions: Magnetic field 336 mT, power 1 mW, amplitude 200, modulation width 0.1 mT, sweep width 10 mT, sweep time 30 sec. Hydroxyl radical scavenging activity was measured by the following equation:

Scavenging activity (%) = (1 − Absorbance of sample/Absorbance of control) × 100

#### 3.5.4. Superoxide Radical Scavenging Activity

Superoxide radical scavenging activity was evaluated by irradiated EDTA system [28]. Briefly, different concentration (0.12–2.00 mg/mL) of sample or standard (ascorbic acid) (20 μL) were mixed with DMPO (0.8 M in 0.1 M PBS of pH 7.4, 20 μL), EDTA (1.6 mM in DW, 20 μL) and riboflavin (0.8 mM in Dw, 20 μL). The mixture was irradiated under a UV lamp at 365 nm for 1 min and then the ESR spectrum was recorded. ESR spectrometer was set at the following conditions: Magnetic field 336 mT, power 10 mW, amplitude 2,500, modulation width 0.1 mT, sweep width 10 mT, sweep time 30 s. Superoxide radical scavenging activity was measured by the following equation:

Scavenging activity (%) = (1 − Absorbance of sample/Absorbance of control) × 100

#### 3.5.5. Metal Chelating Activity

The chelation of ferrous ions by extracts was estimated by method of Dinis *et al.* [29]. Briefly, 2 mM FeCl_2_.4H_2_O (50 μL) was added to different concentrations of the extract (0.12–8.00 mg/mL, 2.5 mL). The reaction was initiated by the addition of 5 mM ferrozine solution (0.2 mL). The mixture was shaken vigorously and left standing at room temperature for 10 min. Absorbance of the solution was then measured at 562 nm against the blank performed in the same way using FeCl_2_ and water. EDTA (3.12–100 µg/mL) served as the positive control and a sample without extract or EDTA served as the negative control. The percentage of inhibition of ferrozine-Fe^2+^ complex formation was calculated using the formula:

Chelating activity (%) = (1 − Absorbance of sample/Absorbance of control) × 100

#### 3.5.6. Inhibition of Lipid Peroxidation Using Thiobarbituric Acid Reactive Substances (TBARS)

Brains were obtained from mice, dissected and homogenized with a Polytron in ice-cold Tris–HCl buffer (20 mM, pH 7.4) to produce a brain tissue homogenate (100 mg/mL) which was centrifuged at 4000 rpm (*vs.* 5000N centrifuge) for 15 min. An aliquot (0.1 mL) of the supernatant was incubated with the different concentrations of the sample (2–0.12 mg/mL; 0.2 mL) in the presence of FeSO_4_ (10 µM; 0.1 mL) and ascorbic acid (0.1 mM; 0.1 mL) at 37 °C for 1 h. The reaction was stopped by the addition of trichloroacetic acid (28% w/v, 0.5 mL), followed by thiobarbituric acid (TBA, 2%, w/v, 0.38 mL), and the mixture was then heated at 80 °C for 20 min. After centrifugation at 3000 rpm for 10 min to remove the precipitated protein, the colour intensity of the malondialdehyde (MDA)–TBA complex in the supernatant was measured by its absorbance at 532 nm [30]. The inhibition ratio (%) was calculated using the following formula:

% Inhibition = (1 − Absorbance of sample/Absorbance of control) × 100

#### 3.5.7. Cell Culture

RAW 264.7, a mouse macrophage cell line, was obtained from the Korean Cell Line Bank (Seoul, Korea) and maintained in DMEM supplemented with 10% heat-inactivated fetal bovine serum (FBS), 100 U/mL penicillin, and 100 mg/mL streptomycin in a humidified atmosphere of 5% CO_2_ at 37 °C. Cell numbers were assessed using a haemocytometer.

#### 3.5.8. Cellular Antioxidant Activity (CAA)

CAA was measured using the method of Wolfe and Liu [21] with various modifications. Raw 264.7 macrophage cells were seeded on 96 well plate at 1 × 10^5^ cells/well. After 16h, cells were treated with various concentrations of the extract for 24 h. Cells were then incubated with DCF-DA (200 µM in DMSO, 10 µL) for 30 min at 37 °C in CO_2_ incubator, followed by addition of H_2_O_2_ (200 µM, 10 µL) and the fluorescence determined (emission at 538 nm and excitation at 485 nm) every 5 min during 1 h at 37 °C. The percent of cellular antioxidant activity (CAA) was calculated as an increase in fluorescent signal between the control and H_2_O_2_ treated cells.

#### 3.5.9. Anti-Hemolysis Activity

The anti-hemolysis activity was assayed according to the method described by Zhang *et al.* [31] with various modifications. Blood was centrifuged at 2500 g and 4 °C for 10 min to separate the red cells from the plasma. The cells were washed twice with PBS (pH 7.4) and centrifuged at 2500 rpm for 2 min. A 5% suspension of red blood cells was used for the test. In each well of a 96-well plate, 100 µL cell suspension, 160 µL AAPH, and 30 µL samples or PBS (blank) were incubated for 3 h at 37 °C. After incubation, PBS (2 mL) was added into the reaction mixture followed by centrifugation at 2500 rpm for 10 min. The absorbance of the supernatant at 540 nm was measured by using spectrophotometer. Percentage of inhibition was calculated by the following equation.

% Inhibition = (1 − Absorbance of sample/Absorbance of control) × 100

### 3.6. Assay of Antioxidant Activity *in Vivo*

#### 3.6.1. Animals and Experimental Design

Antioxidant activity *in vivo* was determined according to the method of Liu *et al.* [22] with some modifications. Female BALB/c mice (weighing 20 ± 2 g, 8 weeks old) were obtained from Orient Bio (Seongnam, Korea). Mice were acclimatized under controlled specific pathogen-free (SPF) conditions for 1 week prior to the start of the experiment. All mice were housed in individual cages and fed with standard laboratory chow in an animal room with 12 h light/dark cycles. The animal study was performed under institutional guidelines [The Institutional Animal Care and Use Committee (IACUC)] at Konkuk University. After adaptation for one week, the mice were randomly divided into five groups (Five mice per group): control group (CG), vitamin C (VC) positive control group (PCG) and three GLBR extract treatment groups. Mice in CG were fed with same volume of water. Mice in PCG were fed with VC (100 mg/kg body weight per day) orally daily using a gavage. Mice in GLBR extract treatment groups were respectively fed with GLBR extract in three different doses (100, 200 and 400 mg/kg body weight per day) by gavage. All groups were performed once daily for 30 consecutive days.

#### 3.6.2. Biochemical Assay

Twenty-four hours after the last drug administration, mice were sacrificed. Blood samples were collected and centrifuged at 3000 rpm at 4 °C for 10 min and the supernatant was collected for analysis. The livers and brains were removed rapidly, washed and homogenized in ice-cold physiological saline to prepare 10% (w/v) homogenate. Then, the homogenate was centrifuged at 3000 rpm at 4 °C for 10 min to remove cellular debris and the supernatant was collected for analysis. Concentration of SOD, CAT and GPx were quantified using kit purchased from Cayman (Ann Arbor, MI, USA). The assays were performed according to the instruction provided in kit manuals.

### 3.7. *In-Vitro* Acetylcholinesterase Assay

The acetyl cholinesterase inhibitory activity of the plant extracts was evaluated by the modified method of Rahman and Choudhary [32]. Briefly, 0.1 M sodium phosphate buffer (pH 8.0, 150 μL), test compound solution (10 μL) and enzyme solution (0.1 units/mL, 20 μL) were mixed and incubated for 15 min at 25 °C. 10 μL of DTNB (10 mM) was then added and reaction was initiated by the addition of substrate (10 μL of ATCI, 14 mM solution). The hydrolysis of the ATCI can be measured by the formation of the coloured product 5-thio-2-nitrobenzoate anion formed by the reaction of DTNB and thiocholine, which is released by the hydrolysis of enzyme. The formation of the colored product was measured at 410 nm wave length after 10min. Tacrine at a final concentration in the assay of 10 µM was used as a positive control. AChE percentage inhibition was calculated by using the equation:

Inhibition activity (%) = (1 − Absorbance of sample/Absorbance of control) × 100

### 3.8. Statistical Analysis

Data were presented as mean ± standard deviation (SD). All analyses were carried out in triplicates. Statistical analyses were performed by one-way analysis of variance (ANOVA). Significant differences between groups were determined at *p* < 0.05. GraphPad Prism 5, Sigma plot 10.0 and Microsoft Excel 2007 were used for the statistical and graphical evaluations.

## 4. Conclusions

To our knowledge this is the first report characterising the phenolic profiles, acetylcholinesterase inhibition, *in vitro* and *in vivo* antioxidant activities of *Ganoderma lucidum* grown on germinated brown rice (GLBR). Results of this study clearly indicated that GLBR extract had significant antioxidant activity against various antioxidant systems *in vitro*. Administration of GLBR extract could significantly enhanced the activities of antioxidant enzymes (SOD, CAT and GPx) in sera, livers and brains of mice. The pronounced antioxidant activity of GLBR extract was possibly due to its high phenolic and flavonoid content. According to the results stated above, it can be concluded that GLBR extract can be used as an accessible source of natural antioxidants and anticholinesterase agents with consequent health benefits.
